# Supplementary material for: Systematic data quality assessment of electronic health record data to evaluate study-specific fitness: Report from the PRESERVE research study
Source: PLOS Digit Health. 2024 Jun 27;3(6):e0000527. doi: 10.1371/journal.pdig.0000527 (PMC11210795; doi:10.1371/journal.pdig.0000527)
Supplement: S1 Table — (DOCX) [file pdig.0000527.s001.docx]

**S1 Table.** Data quality check catalog for DQ1 analysis

| *Check Num* | *Summary* | *Check Type* | *Domain* | *Domain / Variable* | *Check Category* | *Data Quality Probe* | *Clinical Goal* |
| --- | --- | --- | --- | --- | --- | --- | --- |
| 1 | attrition definition | attrition step check | eligibility criteria | attrition variables | completeness | inconsistent trends | clinical care |
| 2 | comparator definitions: total counts | frequency or density distributions | eligibility criteria | attrition variables | conformance | inconsistent trends | diagnostic evaluation |
| 3 | comparator definitions: demographics | frequency or density distributions | eligibility criteria | attrition variables | completeness | anomalous values | epidemiologic distributions |
| 4 | cohort entry year across sites | trends over time | cohort entry date | cohort entry year | completeness | inconsistent trends | epidemiologic distributions |
| 5 | total follow up time: mean and median across institutions, any encounter, in-person encounter | summary statistics | follow up | years of follow up | plausibility | inconsistent trends | clinical care |
| 6 | nephrology specialty as a criterion in the cohort definition; missing values mapped | attrition steps; attribution | specialty | nephrology specialty | conformance | data representation errors | utilization |
| 7 | attrition cohort demographics: ensured demographics were represented | summary statistics | eligibility criteria | demographic variables | conformance | missingness | epidemiologic distributions |
| 8 | serum cystatin: Percent of patients with at least one fact per year stratified by type if applicable | clinical utilization thresholds | laboratory tests | serum cystatin | completeness | data representation errors | clinical care |
| 8 | serum cystatin: Percent of patients with at least one fact per year stratified by type if applicable | trends over time | laboratory tests | serum cystatin | completeness | missingness / data representation | utilization |
| 8 | serum cystatin: Percent of patients with at least one fact per year stratified by type if applicable | variable stratification | laboratory tests | serum cystatin | completeness | missingness / data representation | utilization |
| 9 | serum cystatin: codeset utilization | code utilization | laboratory tests | serum cystatin | plausibility | data representation errors | utilization |
| 10 | serum cystatin: value means and medians | summary statistics | laboratory tests | serum cystatin | plausibility | anomalous values | diagnostic evaluation |
| 10 | serum cystatin: value means and medians | summary statistics | laboratory tests | serum cystatin | concordance | anomalous values | diagnostic evaluation |
| 11 | serum cystatin: potential mismappings by searching raw fields | attribution | laboratory tests | serum cystatin | conformance | data representation errors | diagnostic evaluation |
| 12 | urine creatinine: Percent of patients with at least one fact per year stratified by type if applicable | clinical utilization thresholds | laboratory tests | urine creatinine | completeness | data representation errors | clinical care |
| 12 | urine creatinine: Percent of patients with at least one fact per year stratified by type if applicable | trends over time | laboratory tests | urine creatinine | completeness | missingness / data representation | utilization |
| 12 | urine creatinine: Percent of patients with at least one fact per year stratified by type if applicable | stratification | laboratory tests | urine creatinine | completeness | missingness / data representation | utilization |
| 13 | urine creatinine: codeset utilization | code utilization | laboratory tests | urine creatinine | plausibility | data representation errors | utilization |
| 14 | urine creatinine: value means and medians | summary statistics | laboratory tests | urine creatinine | plausibility | anomalous values | diagnostic evaluation |
| 14 | urine creatinine: value means and medians | summary statistics | laboratory tests | urine creatinine | concordance | anomalous values | diagnostic evaluation |
| 15 | urine creatinine: potential mismappings by searching raw fields | attribution | laboratory tests | urine creatinine | conformance | data representation errors | diagnostic evaluation |
| 16 | urine protein: Percent of patients with at least one fact per year stratified by type if applicable | clinical utilization thresholds | laboratory tests | urine protein | completeness | data representation errors | clinical care |
| 16 | urine protein: Percent of patients with at least one fact per year stratified by type if applicable | trends over time | laboratory tests | urine protein | completeness | missingness / data representation | utilization |
| 16 | urine protein: Percent of patients with at least one fact per year stratified by type if applicable | stratification | laboratory tests | urine protein | completeness | missingness / data representation | utilization |
| 17 | urine protein: codeset utilization | code utilization | laboratory tests | urine protein | plausibility | data representation errors | utilization |
| 18 | urine protein: value means and medians | summary statistics | laboratory tests | urine protein | plausibility | anomalous values | diagnostic evaluation |
| 18 | urine protein: value means and medians | summary statistics | laboratory tests | urine protein | concordance | anomalous values | diagnostic evaluation |
| 19 | urine protein: potential mismappings by searching raw fields | attribution | laboratory tests | urine protein | conformance | data representation errors | diagnostic evaluation |
| 20 | ACE inhibitor: Percent of patients with at least one fact per year stratified by type if applicable | clinical utilization thresholds | medications | ACE inhibitor | completeness | data representation errors | clinical care |
| 20 | ACE inhibitor: Percent of patients with at least one fact per year stratified by type if applicable | trends over time | medications | ACE inhibitor | completeness | missingness / data representation | utilization |
| 20 | ACE inhibitor: Percent of patients with at least one fact per year stratified by type if applicable | stratification | medications | ACE inhibitor | completeness | missingness / data representation | utilization |
| 21 | ACE inhibitor: codeset utilization | code utilization | medications | ACE inhibitor | plausibility | data representation errors | utilization |
| 22 | ACE inhibitor: value means and medians | summary statistics | medications | ACE inhibitor | plausibility | anomalous values | diagnostic evaluation |
| 23 | ACE inhibitor: potential mismappings by searching raw fields | attribution | medications | ACE inhibitor | conformance | data representation errors | diagnostic evaluation |
| 24 | angiotensin receptor blockers: Percent of patients with at least one fact per year stratified by type if applicable | clinical utilization thresholds | medications | angiotensin receptor blockers | completeness | data representation errors | clinical care |
| 24 | angiotensin receptor blockers: Percent of patients with at least one fact per year stratified by type if applicable | trends over time | medications | angiotensin receptor blockers | completeness | missingness / data representation | utilization |
| 24 | angiotensin receptor blockers: Percent of patients with at least one fact per year stratified by type if applicable | stratification | medications | angiotensin receptor blockers | completeness | missingness / data representation | utilization |
| 25 | angiotensin receptor blockers: codeset utilization | code utilization | medications | angiotensin receptor blockers | plausibility | data representation errors | utilization |
| 26 | angiotensin receptor blockers: value means and medians | summary statistics | medications | angiotensin receptor blockers | plausibility | anomalous values | diagnostic evaluation |
| 26 | angiotensin receptor blockers: value means and medians | summary statistics | medications | angiotensin receptor blockers | concordance | anomalous values | diagnostic evaluation |
| 27 | angiotensin receptor blockers: potential mismappings by searching raw fields | attribution | medications | angiotensin receptor blockers | conformance | data representation errors | diagnostic evaluation |
| 28 | beta blockers: Percent of patients with at least one fact per year stratified by type if applicable | clinical utilization thresholds | medications | beta blockers | completeness | data representation errors | clinical care |
| 28 | beta blockers: Percent of patients with at least one fact per year stratified by type if applicable | trends over time | medications | beta blockers | completeness | missingness / data representation | utilization |
| 28 | beta blockers: Percent of patients with at least one fact per year stratified by type if applicable | stratification | medications | beta blockers | completeness | missingness / data representation | utilization |
| 29 | beta blockers: codeset utilization | code utilization | medications | beta blockers | plausibility | data representation errors | utilization |
| 30 | beta blockers: value means and medians | summary statistics | medications | beta blockers | plausibility | anomalous values | diagnostic evaluation |
| 30 | beta blockers: value means and medians | summary statistics | medications | beta blockers | concordance | anomalous values | diagnostic evaluation |
| 31 | beta blockers: potential mismappings by searching raw fields | attribution | medications | beta blockers | conformance | data representation errors | diagnostic evaluation |
| 32 | calcium channel blockers: Percent of patients with at least one fact per year stratified by type if applicable | clinical utilization thresholds | medications | calcium channel blockers | completeness | data representation errors | clinical care |
| 32 | calcium channel blockers: Percent of patients with at least one fact per year stratified by type if applicable | trends over time | medications | calcium channel blockers | completeness | missingness / data representation | utilization |
| 32 | calcium channel blockers: Percent of patients with at least one fact per year stratified by type if applicable | stratification | medications | calcium channel blockers | completeness | missingness / data representation | utilization |
| 33 | calcium channel blockers: codeset utilization | code utilization | medications | calcium channel blockers | plausibility | data representation errors | utilization |
| 34 | calcium channel blockers: value means and medians | summary statistics | medications | calcium channel blockers | plausibility | anomalous values | diagnostic evaluation |
| 34 | calcium channel blockers: value means and medians | summary statistics | medications | calcium channel blockers | concordance | anomalous values | diagnostic evaluation |
| 35 | calcium channel blockers: potential mismappings by searching raw fields | attribution | medications | calcium channel blockers | conformance | data representation errors | diagnostic evaluation |
| 36 | loop diuretics: Percent of patients with at least one fact per year stratified by type if applicable | clinical utilization thresholds | medications | loop diuretics | completeness | data representation errors | clinical care |
| 36 | loop diuretics: Percent of patients with at least one fact per year stratified by type if applicable | trends over time | medications | loop diuretics | completeness | missingness / data representation | utilization |
| 36 | loop diuretics: Percent of patients with at least one fact per year stratified by type if applicable | stratification | medications | loop diuretics | completeness | missingness / data representation | utilization |
| 37 | loop diuretics: codeset utilization | code utilization | medications | loop diuretics | plausibility | data representation errors | utilization |
| 38 | loop diuretics: value means and medians | summary statistics | medications | loop diuretics | plausibility | anomalous values | diagnostic evaluation |
| 38 | loop diuretics: value means and medians | summary statistics | medications | loop diuretics | concordance | anomalous values | diagnostic evaluation |
| 39 | loop diuretics: potential mismappings by searching raw fields | attribution | medications | loop diuretics | conformance | data representation errors | diagnostic evaluation |
| 40 | thiazide diuretics: Percent of patients with at least one fact per year stratified by type if applicable | clinical utilization thresholds | medications | thiazide diuretics | completeness | data representation errors | clinical care |
| 40 | thiazide diuretics: Percent of patients with at least one fact per year stratified by type if applicable | trends over time | medications | thiazide diuretics | completeness | missingness / data representation | utilization |
| 40 | thiazide diuretics: Percent of patients with at least one fact per year stratified by type if applicable | stratification | medications | thiazide diuretics | completeness | missingness / data representation | utilization |
| 41 | thiazide diuretics: codeset utilization | code utilization | medications | thiazide diuretics | plausibility | data representation errors | utilization |
| 42 | thiazide diuretics: value means and medians | summary statistics | medications | thiazide diuretics | plausibility | anomalous values | diagnostic evaluation |
| 42 | thiazide diuretics: value means and medians | summary statistics | medications | thiazide diuretics | concordance | anomalous values | diagnostic evaluation |
| 43 | thiazide diuretics: potential mismappings by searching raw fields | attribution | medications | thiazide diuretics | conformance | data representation errors | diagnostic evaluation |
| 44 | kidney transplant px: Percent of patients with at least one fact per year stratified by type if applicable | clinical utilization thresholds | procedures | kidney transplant | completeness | data representation errors | clinical care |
| 44 | kidney transplant px: Percent of patients with at least one fact per year stratified by type if applicable | trends over time | procedures | kidney transplant | completeness | missingness / data representation | utilization |
| 44 | kidney transplant px: Percent of patients with at least one fact per year stratified by type if applicable | stratification | procedures | kidney transplant | completeness | missingness / data representation | utilization |
| 45 | kidney transplant px: codeset utilization | code utilization | procedures | kidney transplant | plausibility | data representation errors | utilization |
| 46 | kidney transplant px: value means and medians | summary statistics | procedures | kidney transplant | plausibility | anomalous values | diagnostic evaluation |
| 46 | kidney transplant px: value means and medians | summary statistics | procedures | kidney transplant | concordance | anomalous values | diagnostic evaluation |
| 47 | kidney transplant px: potential mismappings by searching raw fields | attribution | procedures | kidney transplant | conformance | data representation errors | diagnostic evaluation |
| 48 | kidney biopsy px: Percent of patients with at least one fact per year stratified by type if applicable | clinical utilization thresholds | procedures | kidney biopsy | completeness | data representation errors | clinical care |
| 48 | kidney biopsy px: Percent of patients with at least one fact per year stratified by type if applicable | trends over time | procedures | kidney biopsy | completeness | missingness / data representation | utilization |
| 48 | kidney biopsy px: Percent of patients with at least one fact per year stratified by type if applicable | stratification | procedures | kidney biopsy | completeness | missingness / data representation | utilization |
| 49 | kidney biopsy px: codeset utilization | code utilization | procedures | kidney biopsy | plausibility | data representation errors | utilization |
| 50 | kidney biopsy px: value means and medians | summary statistics | procedures | kidney biopsy | plausibility | anomalous values | diagnostic evaluation |
| 51 | kidney biopsy px: potential mismappings by searching raw fields | attribution | procedures | kidney biopsy | conformance | data representation errors | diagnostic evaluation |
| 52 | kidney transplant dx: Percent of patients with at least one fact per year stratified by type if applicable | clinical utilization thresholds | diagnoses | kidney transplant | completeness | data representation errors | clinical care |
| 52 | kidney transplant dx: Percent of patients with at least one fact per year stratified by type if applicable | trends over time | diagnoses | kidney transplant | completeness | missingness / data representation | utilization |
| 52 | kidney transplant dx: Percent of patients with at least one fact per year stratified by type if applicable | stratification | diagnoses | kidney transplant | completeness | missingness / data representation | utilization |
| 53 | kidney transplant dx: codeset utilization | code utilization | diagnoses | kidney transplant | plausibility | data representation errors | utilization |
| 54 | kidney transplant dx: value means and medians | summary statistics | diagnoses | kidney transplant | plausibility | anomalous values | diagnostic evaluation |
| 54 | kidney transplant dx: value means and medians | summary statistics | diagnoses | kidney transplant | concordance | anomalous values | diagnostic evaluation |
| 55 | kidney transplant dx: potential mismappings by searching raw fields | attribution | diagnoses | kidney transplant | conformance | data representation errors | diagnostic evaluation |
| 56 | CKD 2 or 3 dx: Percent of patients with at least one fact per year stratified by type if applicable | clinical utilization thresholds | diagnoses | CKD 2 or 3 | completeness | data representation errors | clinical care |
| 56 | CKD 2 or 3 dx: Percent of patients with at least one fact per year stratified by type if applicable | trends over time | diagnoses | CKD 2 or 4 | completeness | missingness / data representation | utilization |
| 56 | CKD 2 or 3 dx: Percent of patients with at least one fact per year stratified by type if applicable | stratification | diagnoses | CKD 2 or 5 | completeness | missingness / data representation | utilization |
| 57 | CKD 2 or 3 dx: codeset utilization | code utilization | diagnoses | CKD 2 or 6 | plausibility | data representation errors | utilization |
| 58 | CKD 2 or 3 dx: value means and medians | summary statistics | diagnoses | CKD 2 or 7 | plausibility | anomalous values | diagnostic evaluation |
| 58 | CKD 2 or 3 dx: value means and medians | summary statistics | diagnoses | CKD 2 or 7 | concordance | anomalous values | diagnostic evaluation |
| 59 | CKD 2 or 3 dx: potential mismappings by searching raw fields | attribution | diagnoses | CKD 2 or 8 | conformance | data representation errors | diagnostic evaluation |
| 60 | serum creatinine: Percent of patients with at least one fact per year stratified by type if applicable | clinical utilization thresholds | laboratory tests | serum creatinine | completeness | data representation errors | clinical care |
| 60 | serum creatinine: Percent of patients with at least one fact per year stratified by type if applicable | trends over time | laboratory tests | serum creatinine | completeness | missingness / data representation | utilization |
| 60 | serum creatinine: Percent of patients with at least one fact per year stratified by type if applicable | stratification | laboratory tests | serum creatinine | completeness | missingness / data representation | utilization |
| 61 | serum creatinine: codeset utilization | code utilization | laboratory tests | serum creatinine | plausibility | data representation errors | utilization |
| 62 | serum creatinine: value means and medians | summary statistics | laboratory tests | serum creatinine | plausibility | anomalous values | diagnostic evaluation |
| 62 | serum creatinine: value means and medians | summary statistics | laboratory tests | serum creatinine | concordance | anomalous values | diagnostic evaluation |
| 63 | serum creatinine: potential mismappings by searching raw fields | attribution | laboratory tests | serum creatinine | conformance | data representation errors | diagnostic evaluation |
| 64 | sbp: percent of patients with at least one measurements per year | clinical utilization thresholds | blood pressure | systolic bp | completeness | data representation errors | clinical care |
| 64 | sbp: percent of patients with at least one measurements per year | trends over time | blood pressure | systolic bp | completeness | missingness | utilization |
| 65 | sbp: measurements per person over time | clinical facts per patient | blood pressure | systolic bp | completeness | missingness | utilization |
| 65 | sbp: measurements per person over time | trends over time | blood pressure | systolic bp | completeness | inconsistent trends | utilization |
| 66 | sbp: mean value for age | trends over time | blood pressure | systolic bp | completeness | inconsistent trends | diagnostic evaluation |
| 66 | sbp: mean value for age | summary statistics | blood pressure | systolic bp | plausibility | anomalous values | epidemiologic distributions |
| 67 | sbp: violin plots of values, with weighted means | frequency or density distributions | blood pressure | systolic bp | plausibility | anomalous values | diagnostic evaluation |
| 68 | dbp: percent of patients with at least one measurements per year | clinical utilization thresholds | blood pressure | diastolic bp | completeness | data representation errors | clinical care |
| 68 | dbp: percent of patients with at least one measurements per year | trends over time | blood pressure | diastolic bp | completeness | missingness | utilization |
| 69 | dbp: measurements per person over time | clinical facts per patient | blood pressure | diastolic bp | completeness | missingness | utilization |
| 69 | dbp: measurements per person over time | trends over time | blood pressure | diastolic bp | completeness | inconsistent trends | utilization |
| 70 | dbp: mean value for age | trends over time | blood pressure | diastolic bp | completeness | inconsistent trends | diagnostic evaluation |
| 70 | dbp: mean value for age | summary statistics | blood pressure | diastolic bp | completeness | anomalous values | epidemiologic distributions |
| 71 | dbp: violin plots of values, with weighted means | frequency or density distributions | blood pressure | diastolic bp | plausibility | anomalous values | diagnostic evaluation |
| 72 | weights: percent of patients with at least one measurement per year | clinical utilization thresholds | anthropo-metrics | body weight | completeness | data representation errors | clinical care |
| 72 | weights: percent of patients with at least one measurement per year | trends over time | anthropo-metrics | body weight | completeness | missingness | utilization |
| 73 | weights: measurements per person over time | clinical facts per patient | anthropo-metrics | body weight | completeness | inconsistent trends | utilization |
| 73 | weights: measurements per person over time | trends over time | anthropo-metrics | body weight | completeness | inconsistent trends | utilization |
| 74 | weights: mean value over time | trends over time | anthropo-metrics | body weight | completeness | inconsistent trends | diagnostic evaluation |
| 74 | weights: mean value over time | summary statistics | anthropo-metrics | body weight | plausibility | anomalous values | epidemiologic distributions |
| 75 | weights: comparison of mean/median measurements | summary statistics | anthropo-metrics | body weight | concordance | anomalous values | diagnostic evaluation |
| 76 | heights: percent of patients with at least one measurement per year | clinical utilization thresholds | anthropo-metrics | body height | completeness | data representation errors | clinical care |
| 76 | heights: percent of patients with at least one measurement per year | trends over time | anthropo-metrics | body height | completeness | missingness | utilization |
| 77 | heights: measurements per person over time | clinical facts per patient | anthropo-metrics | body height | completeness | inconsistent trends | utilization |
| 77 | heights: measurements per person over time | trends over time | anthropo-metrics | body height | completeness | inconsistent trends | utilization |
| 78 | heights: mean value over time | summary statistics | anthropo-metrics | body height | completeness | inconsistent trends | diagnostic evaluation |
| 78 | heights: mean value over time | clinical fact temporal relationships | anthropo-metrics | body height | plausibility | anomalous values | epidemiologic distributions |
| 79 | heights: comparison of mean/median measurements | summary statistics | anthropo-metrics | body height | concordance | anomalous values | diagnostic evaluation |
