# Supplementary material for: Systematic data quality assessment of electronic health record data to evaluate study-specific fitness: Report from the PRESERVE research study
Source: PLOS Digit Health. 2024 Jun 27;3(6):e0000527. doi: 10.1371/journal.pdig.0000527 (PMC11210795; doi:10.1371/journal.pdig.0000527)
Supplement: S2 Table — (DOCX) [file pdig.0000527.s002.docx]

**S2 Table.** Data quality check catalog for DQ2 analysis

| *Check Num* | *Summary* | *Check Type* | *Domain* | *Domain / Variable* | *Check Category* | *Data Quality Probe* | *Clinical Goal* |
| --- | --- | --- | --- | --- | --- | --- | --- |
| 1 | attrition definition | attrition step check | eligibility criteria | attrition variables | completeness | inconsistent trends | clinical care |
| 2 | demographic breakdown | summary statistics | eligibility criteria | demographic variables | plausibility | anomalous values | epidemiologic distributions |
| 3 | cohort entry year across sites | trends over time | cohort entry date | cohort entry year | completeness | inconsistent trends | epidemiologic distributions |
| 4 | average visits per person, before and after CED, stratified by type | temporal relationships | visits | visit utilization; cohort entry year | completeness | anomalous event sequencing | diagnostic evaluation |
| 4 | average visits per person, before and after CED, stratified by type | clinical facts per patient | visits | visit utilization; cohort entry year | consistency | anomalous values | utilization |
| 4 | average visits per person, before and after CED, stratified by type | variable stratifications | visits | visit utilization; cohort entry year | completeness | anomalous values | clinical care |
| 4 | average visits per person, before and after CED, stratified by type | clinical specialty utilization | visits | visit utilization; cohort entry year | plausibility | inconsistent trends | utilization |
| 5 | percent cohort with encounter per year, stratified by type | variable stratifications | visits | visit utilization | completeness | inconsistent trends | utilization |
| 5 | percent cohort with encounter per year, stratified by type | clinical utilization thresholds | visits | visit utilization | completeness | anomalous values | clinical care |
| 5 | percent cohort with encounter per year, stratified by type | trends over time | visits | visit utilization | completeness | inconsistent trends | utilization |
| 6 | percent encounters not associated with clinical facts | variable stratifications | clinical facts | multiple clinical domains | completeness | inconsistent trends | utilization |
| 6 | percent encounters not associated with clinical facts | clinical utilization thresholds | clinical facts | multiple clinical domains | completeness | anomalous values | clinical care |
| 6 | percent encounters not associated with clinical facts | trends over time | clinical facts | multiple clinical domains | completeness | inconsistent trends | utilization |
| 7 | diagnoses per patient, stratified by type | clinical facts per patient | diagnoses | diagnoses | completeness | anomalous values | clinical care |
| 7 | diagnoses per patient, stratified by type | trends over time | diagnoses | diagnoses | completeness | inconsistent trends | utilization |
| 7 | diagnoses per patient, stratified by type | variable stratifications | diagnoses | diagnoses | completeness | anomalous values | clinical care |
| 8 | medications per patient, stratified by type | clinical facts per patient | medications | medications | completeness | anomalous values | clinical care |
| 8 | medications per patient, stratified by type | trends over time | medications | medications | completeness | inconsistent trends | utilization |
| 8 | medications per patient, stratified by type | variable stratifications | medications | medications | completeness | anomalous values | clinical care |
| 9 | vitals per patient | clinical facts per patient | vital signs | vitals | completeness | anomalous values | clinical care |
| 9 | vitals per patient | trends over time | vital signs | vitals | completeness | inconsistent trends | utilization |
| 9 | vitals per patient | variable stratifications | vital signs | vitals | completeness | anomalous values | clinical care |
| 10 | procedures per patient | clinical facts per patient | procedures | procedures | completeness | anomalous values | clinical care |
| 10 | procedures per patient | trends over time | procedures | procedures | completeness | inconsistent trends | utilization |
| 10 | procedures per patient | variable stratifications | procedures | procedures | completeness | anomalous values | clinical care |
| 11 | labs per patient | clinical facts per patient | laboratory tests | labs | completeness | anomalous values | clinical care |
| 11 | labs per patient | trends over time | laboratory tests | labs | completeness | inconsistent trends | utilization |
| 11 | labs per patient | variable stratifications | laboratory tests | labs | completeness | anomalous values | clinical care |
| 12 | nephrology visit type proportion of total visits | variable stratifications | visits | nephrology visits | completeness | anomalous values | clinical care |
| 12 | nephrology visit type proportion of total visits | clinical utilization thresholds | visits | nephrology visits | plausibility | anomalous values | clinical care |
| 12 | nephrology visit type proportion of total visits | clinical specialty utilization | visits | nephrology visits | concordance | anomalous values | clinical care |
| 12 | nephrology visit type proportion of total visits | clinical specialty utilization | visits | nephrology visits | plausibility | anomalous values | clinical care |
| 13 | nephrology visit type proportion of total visits | trends over time | visits | nephrology visits | completeness | inconsistent trends | utilization |
| 13 | percent of encounters associated with nephrology provider or care site | code utilizatioin | specialty | nephrology visits | conformance | data representation error | utilization |
| 13 | percent of encounters associated with nephrology provider or care site | code utilizatioin | specialty | nephrology visits | concordance | data representation error | utilization |
| 14 | unique nephrology facility IDs, stratified by visit type | summary statistics | specialty | nephrology visits | conformance | data representation error | utilization |
| 15 | antihypertensive medications: timing from cohort entry | clinical fact relationships | medications | antihyper-tensive medications; cohort entry date | plausibility | inconsistent trends | diagnostic evaluation |
| 16 | antihypertensive medications: proportion of patients on | clinical utilization thresholds | medications | antihyper-tensive medications; cohort entry date | completeness | anomalous values | diagnostic evaluation |
| 16 | antihypertensive medications: proportion of patients on | clinical utilization thresholds | medications | antihyper-tensive medications; cohort entry date | concordance | anomalous values | diagnostic evaluation |
| 17 | antihypertensive medications: before and after cohort entry | fact proportions | medications | antihyper-tensive medications; cohort entry date | completeness | anomalous event sequencing | diagnostic evaluation |
| 18 | eGFR: median before and after CED, across calendar year | temporal relationships | laboratory tests | eGFR; cohort entry year | plausibility | anomalous event sequencing | clinical care |
| 18 | eGFR: median before and after CED, across calendar year | trends over time | laboratory tests | eGFR; cohort entry year | completeness | inconsistent trends | clinical care |
| 19 | eGFR: median before and after CED, years from cohort entry | trends over time | laboratory tests | eGFR; cohort entry date | consistency | anomalous event sequencing | clinical care |
| 20 | eGFR: height and scr measurements | clinical fact relationships | anthropometrics | heights; weights | concordance | missingness | utilization |
| 20 | eGFR: height and scr measurements | clinical fact relationships | laboratory tests | heights; weights | concordance | missingness | utilization |
| 21 | eGFR: measurements per year, in patients with at least one measurement | trends over time | derviations | eGFR | completeness | inconsistent trends | utilization |
| 21 | eGFR: measurements per year, in patients with at least one measurement | clinical facts per patient | derviations | eGFR | completeness | anomalous values | clinical care |
| 22 | eGFR: proportion with >= 1 low eGFR with varying thresholds | clinical thresholds | derviations | eGFR | consistency | inconsistent trends | diagnostic evaluation |
| 22 | eGFR: proportion with >= 1 low eGFR with varying thresholds | attribution | derviations | eGFR | plausibility | anomalous values | diagnostic evaluation |
| 23 | eGFR: median proportion of low eGFR for patients with 1 or more value | attribution | derviations | eGFR | plausibility | anomalous values | diagnostic evaluation |
| 23 | eGFR: median proportion of low eGFR for patients with 1 or more value | clinical thresholds | derviations | eGFR | consistency | inconsistent trends | diagnostic evaluation |
| 24 | eGFR: median (iqr) of patients with eGFR below 30 for patients with >= 1 below 30 | clinical thresholds | derviations | eGFR | consistency | inconsistent trends | diagnostic evaluation |
| 25 | urine protein: number before and after CED | temporal relationships | laboratory tests | urine protein; cohort entry year | plausibility | anomalous event sequencing | clinical care |
| 26 | urine protein: qualitative or quantitative labs | code utilization | laboratory tests | urine protein; cohort entry year | conformance | data representation error | clinical care |
| 27 | urine protein: measurements per patient | clinical facts per patient | laboratory tests | urine protein | completeness | anomalous values | utilization |
| 28 | urine protein: mapped qualitative and quantitative results | code utilization | laboratory tests | urine protein | conformance | data representation error | utilization |
| 29 | urine protein: unit mappings | frequency or density distributions | laboratory tests | urine protein | conformance | data representation error | utilization |
| 30 | urine protein: qualitative distribution of results | frequency or density distributions | laboratory tests | urine protein | plausibility | anomalous values | clinical care |
| 31 | urine protein: quantitative distribution of results | frequency or density distributions | laboratory tests | urine protein | plausibility | anomalous values | clinical care |
| 32 | chronic dialysis: presence of expected codes | code utilization | laboratory tests | chronic dialysis | conformance | data representation error | clinical care |
| 33 | chronic dialysis: frequency of expected codes | frequency or density distributions | laboratory tests | chronic dialysis | plausibility | anomalous values | clinical care |
| 34 | kidney transplant: presence of expected codes | code utilization | procedures | kidney transplant | conformance | data representation error | clinical care |
| 35 | kidney transplant: frequency of expected codes | frequency or density distributions | procedures | kidney transplant | plausibility | anomalous values | clinical care |
| 36 | ABPM: presence of expected codes | code utilization | procedures | ABPM | conformance | data representation error | clinical care |
| 37 | ABPM: frequency of expected codes | frequency or density distributions | procedures | ABPM | plausibility | anomalous values | clinical care |
| 38 | adverse events: proportion of patients experiencing (asthma) | summary statistics | diagnoses | asthma | plausibility | anomalous values | diagnostic evaluation |
| 39 | adverse events: proportion of patients experiencing (cough) | summary statistics | diagnoses | cough | plausibility | anomalous values | diagnostic evaluation |
| 40 | adverse events: proportion of patients experiencing (depression) | summary statistics | diagnoses | depression | plausibility | anomalous values | diagnostic evaluation |
| 41 | adverse events: proportion of patients experiencing (dizziness) | summary statistics | diagnoses | dizziness | plausibility | anomalous values | diagnostic evaluation |
| 42 | adverse events: proportion of patients experiencing (fatigue) | summary statistics | diagnoses | fatigue | plausibility | anomalous values | diagnostic evaluation |
| 43 | adverse events: proportion of patients experiencing (hypotension) | summary statistics | diagnoses | hypotension | plausibility | anomalous values | diagnostic evaluation |
| 44 | adverse events: proportion of patients experiencing (pericarditis) | summary statistics | diagnoses | pericarditis | plausibility | anomalous values | diagnostic evaluation |
| 45 | adverse events (all): proportion and distribution of adverse events | summary statistics | diagnoses | summary adverse events | plausibility | anomalous values | diagnostic evaluation |
| 46 | blood pressure: measurements present on same day | clinical fact relationships | vital signs | systolic BP; diastolic BP | concordance | missingness | clinical care |
| 47 | blood pressure values: systolic and diastolic correlation | clinical correlations | vital signs | systolic BP; diastolic BP | consistency | inconsistent trends | clinical care |
| 48 | blood pressure values summary (systolic): outliers | summary statistics | vital signs | systolic BP | plausibility | anomalous values | diagnostic evaluation |
| 49 | blood pressure values distribution (systolic): distributions | frequency or density distributions | vital signs | systolic BP | plausibility | anomalous values | diagnostic evaluation |
| 50 | blood pressure values summary (diastolic): outliers | summary statistics | vital signs | diastolic BP | plausibility | anomalous values | diagnostic evaluation |
| 51 | blood pressure values distribution (diastolic): distributions | frequency or density distributions | vital signs | diastolic BP | plausibility | anomalous values | diagnostic evaluation |
| 52 | blood pressure values summary statistics: systolic | summary statistics | vital signs | systolic BP | plausibility | anomalous values | epidemiologic distributions |
| 53 | blood pressure values before and after cohort entry: systolic | clinical fact relationships | vital signs | systolic BP | consistency | inconsistent trends | diagnostic evaluation |
| 54 | blood pressure values summary statistics: diastolic | summary statistics | vital signs | diastolic BP | plausibility | anomalous values | epidemiologic distributions |
| 55 | blood pressure values before and after cohort entry: diastolic | clinical fact relationships | vital signs | diastolic BP | consistency | inconsistent trends | diagnostic evaluation |
| 56 | blood pressure measurements per person | clinical facts per patient | vital signs | BP (any) | completeness | anomalous values | utilization |
| 57 | blood pressure measurements per person: before/after cohort entry | clinical fact relationships | vital signs | BP (any) | consistency | inconsistent trends | clinical care |
| 57 | blood pressure measurements per person: before/after cohort entry | clinical fact relationships | cohort entry date | BP (any) | consistency | inconsistent trends | clinical care |
| 58 | geographic variables: proportion with ZIP5 | summary statistics | geographic variables | 5-digit ZIP | completeness | missingness | epidemiologic distributions |
| 59 | geographic variables: proportion with ZIP9 | summary statistics | geographic variables | 9-digit ZIP | completeness | missingness | epidemiologic distributions |
| 60 | geographic variables: proportion with census tract | summary statistics | geographic variables | census tract | completeness | missingness | epidemiologic distributions |
| 61 | geographic variables: proportion with census block group | summary statistics | geographic variables | census block group | completeness | missingness | epidemiologic distributions |
| 62 | geographic variables: address history ZIP5 | clinical facts per patient | geographic variables | address history; 5-digit ZIP | completeness | missingness | epidemiologic distributions |
| 63 | geographic variables: address history with ZIP9 | clinical facts per patient | geographic variables | address history; 9-digit ZIP | completeness | missingness | epidemiologic distributions |
| 64 | geographic variables: address history with census tract | clinical facts per patient | geographic variables | address history; census tract | completeness | missingness | epidemiologic distributions |
| 65 | geographic variables: address history with census block group | clinical facts per patient | geographic variables | address history; census block group | completeness | missingness | epidemiologic distributions |
