# Supplementary material for: Systematic data quality assessment of electronic health record data to evaluate study-specific fitness: Report from the PRESERVE research study
Source: PLOS Digit Health. 2024 Jun 27;3(6):e0000527. doi: 10.1371/journal.pdig.0000527 (PMC11210795; doi:10.1371/journal.pdig.0000527)
Supplement: S3 Table — (DOCX) [file pdig.0000527.s003.docx]

**S3 Table.** Considerations for issue remediation

| *Missingness, complete or near-complete* |
| --- |
| Data not recorded at source |
| Misidentification of location in source systems |
| Different coding system in use |
| ETL inclusion error |
| *Missingness, partial* |
| Variable data capture – consider secular trends in deployment or terminology |
| ETL constraint error |
| Incomplete selection criteria (*e.g.*, codeset) |
| *Atypical code distribution* |
| *Atypical numeric distribution* |
| Population or case mix difference |
| Coding practice variation |
| Incomplete data capture across population (*e.g.,* access, specialty practice) |
| ETL code mapping error |
| *Atypical numeric distribution* |
| ETL unit mapping/conversion error |
| *Discordant values* |
| *High Counts* |
| *Low Counts* |
| Incomplete data capture |
| ETL inclusion error |
| Incomplete selection criteria (*e.g.*, codeset) |
| *High spike* |
| *Low Spike* |
| Secular changes in code usage or selection criteria |
| Population change (*e.g.*, entry of new health system) |
| Clinical system deployment |
| Major event (*e.g.*, pandemic, public event) |
| *Mapping error* |
| Secular change in code usage |
| Invalid cross-walk |
| Subject matter error |
| *Outlier values* |
| ETL unit mapping/conversion error |
| Extreme value as event marker (*e.g.*, unavailable drug dose) |
