# Supplementary material for: Systematic data quality assessment of electronic health record data to evaluate study-specific fitness: Report from the PRESERVE research study
Source: PLOS Digit Health. 2024 Jun 27;3(6):e0000527. doi: 10.1371/journal.pdig.0000527 (PMC11210795; doi:10.1371/journal.pdig.0000527)
Supplement: Attachment — Submitted filename: Recommendations SSDQA.pdf [file pdig.0000527.s005.pdf]

Recommendation

Major Revision

The manuscript effectively demonstrates the application of systematic data quality assessment in a real-world research setting. The thorough evaluation of fitness for study-specific requirements is commendable and highlights the importance of addressing data quality issues in secondary analyses of clinical data.

Besides, the manuscript provides a comprehensive analysis of the application of a study-specific data quality assessment (SSDQA) framework in the context of the PRESERVE study. However, it has several limitations and areas for improvement. These include limited generalizability, resource intensiveness, incomplete evaluation, lack of longitudinal analysis, and absence of comparative analysis.

The manuscript lacks a thorough discussion of existing research on SSDQA frameworks in the context of observational studies using electronic health record (EHR) data. A more comprehensive review of the literature could help contextualize the study's contributions and identify gaps in current knowledge or methodologies. Comparing the SSDQA framework with existing approaches in the literature would help evaluate its novelty and effectiveness.

Validation and benchmarking of the framework against established standards or best practices in data quality assessment would enhance the credibility and reliability of the findings. Future directions for the SSDQA framework could be discussed, including potential extensions or refinements, opportunities for further empirical testing, and cross-disciplinary insights from related disciplines such as epidemiology, biostatistics, and health services research.

Incorporating insights from related disciplines like epidemiology, biostatistics, and health services research could enrich the discussion and broaden the study's relevance to a wider audience. Addressing these limitations could enhance the manuscript's contribution to the field by providing a more nuanced understanding of the SSDQA framework's effectiveness and applicability in observational health data analysis.
