# Supplementary material for: Systematic data quality assessment of electronic health record data to evaluate study-specific fitness: Report from the PRESERVE research study
Source: PLOS Digit Health. 2024 Jun 27;3(6):e0000527. doi: 10.1371/journal.pdig.0000527 (PMC11210795; doi:10.1371/journal.pdig.0000527)
Supplement: Attachment — Submitted filename: Reviewer_response.docx [file pdig.0000527.s004.docx]

Responses to Reviewers

*Reviewer #1: This manuscript offers an evaluation of fitness of data for intended research use that was performed via a 2-round data quality assessment (DQA) of a 16-site EHR-based study of chronic kidney disease. Unfortunately, the writing and organization of the content in this manuscript was very difficult to follow. There are details that appear inconsistent, calling into question the rigor of the work and robusticity of findings reported. The methods were inadequately described (e.g., while several places refer to 2 rounds of DQA, on line 445 a third round is indicated). This might be attributable to overreliance on Razzaghi et al. 2022 when details must be addressed in the current manuscript.*

*While this 16-site study might be an illustrative example for how a DQA has been performed and enable an enlightening discussion of the benefits that arise from such processes being performed, the manuscript itself (with its inconsistencies in reporting, ambiguity of methodology, and organizational problems) does not clarify how and to what extent data quality issues were resolved in this instance or how they could be in other applications of a DQA. For example, the abstract contains aspects that are not developed in the manuscript (e.g., how two institutions’ exclusion was avoided; how exactly data problems were avoided once identified; in what regard multidisciplinary teams are advantageous; etc.).*

*I've included more specific feedback in the hopes you find it constructive when revising the manuscript:*

*1. In both Round 1 DQ and Round 2 DQ, how were the designations (i.e., urgent, high, medium, and low priority) made and by whom? How were themes identified? Was there a qualitative code book used by researchers? If more than one researcher made the designation, how were discrepancies handled? If the “related to” sentences appearing on p18 are about the data quality designation, it’s unclear why that is being discussed in a section labeled “Data Quality Remediation” (which implies corrective action following identification of a data quality issue). In sum, the methods are not adequately explained or, at best, are oddly relegated to the end of the paper. For example, it is only at line 445 that readers suddenly see there was a Round 3 performed. The two supplemental tables of “catalogs of checks and accompanying annotation” are not clearly explained as to how they were created, applied, or interpreted.*

**Thank you for the comment, which touches on several areas of the paper. We made significant revisions to the manuscript to address these concerns. To summarize:**

**Prioritization**

- **We moved description of priorities to the Methods section and included the process for designating as urgent, high, medium, and low priority, which can be found under the subsection “SSDQA Evaluation”.**
- **We discuss priority designation in the Results as examples of data quality issues that fell into each category. The purpose of the prioritization was specifically to help analysts with responding to the feedback we provided to them, rather than overarching assertions about the issues outside of this context.**
- **We added to the Results section a figure showing issue remediation by priority designation.**

**Process Clarification**

- **We believed we were conforming to journal submission requirements by placing the Methods at the end of the manuscript. However, we agree with the reviewer that for this particular study, it was difficult to follow the Results without reading the Methods first. We have therefore moved the Methods section up before the Results section.**
- **To clarify the different rounds of data quality, we added a section in the Methods section called “Check Sequencing”.**
- **We have added a “SSDQA Evaluation” section, where issue catalog is described in more detail. The process of identifying themes was also placed in the Methods section.**

**Cataloging and Theme Identification**

- **We have described in more depth both the check and issue catalogs. All columns in both catalogs are described in the manuscript. These catalogs serve the purpose of organizing checks, as well as the issues or problems that we identified from the application of the checks. We want to stress that in other data quality programs, cataloging is rarely performed beyond very general terms (e.g., *completeness, conformance, plausibility*), and to our knowledge, we are among the first to assign additional meaningful metadata to issues. We identified a need for these catalogs during our process and created these catalogs as a tool of communication, but also as a step for reproducibility in the future.**
- **We have described in Methods the process for theme assignment. We held meetings to ensure that issues could be assigned to themes, and the lead and senior author concurred on the final assignment of themes. The purpose of this categorization was to summarize data quality findings, and to provide a meaningful interpretation for the types of data quality issues that can affect studies for those less familiar with data quality terms and metadata.**

*2. Fig. 1 displays only 12 sites, Fig. 2, Fig. 3, Fig. 6, and Fig. 7 display only 15 sites, and Fig. 4 displays 14 sites; however, the description of the PRESERVE study is that there are 16 sites. Only Fig. 5 displays 16 sites. Why is there a discrepancy? This should be clarified somewhere (whether in a figure caption or discussion appearing in the main text).*

**We had originally limited the figures to sites that had significant data the check being shown, in the hope of making the figures less cluttered, but we can see the reviewer’s point about confusion due to incomplete representation. We have remade the figures to show a consistent 15 sites across all, and removed a 16^th^ site that withdrew from the study during the DQ process.**

*3. Table 2 has confusing column headers and content - particularly when one recognizes that readers will not yet have seen the methods buried at the bottom of the manuscript.*

**In addition to moving up Methods, we have addressed this comment by adding more explanatory text to the manuscript about the contents in the table. That table is now Table 4 in the manuscript, and discussed in the Results section under the subheading “Data Quality Remediation and Improvement”.**

*4. What do the authors mean by “misclassified ethnicity” at line 243 p19? Misclassified by whom, in what way, and when? How are population identifiers and their inconsistencies addressed? While the 2023 NASEM report on population identifiers was focused on genetic and genomic research uses, the definitional clarifications are relevant and important considerations with regard to EHR data’s fit-for-purpose as well, particularly when one acknowledges that ethnic identity (if you are talking about patient self-reported ethnicity) develops and changes over individuals’ lifetimes and also, distinctly, the social constructs change over time (see, e.g., the PEW Research chart of Feb. 6, 2020 clarifying how the US Census has altered its handling of race and ethnicity over time at* [*https://www.pewresearch.org/interactives/what-census-calls-us/)*](%20https://www.pewresearch.org/interactives/what-census-calls-us/))*. For example, did you examine how, precisely, Meaningful Use Stage 2 (which involved the 1997 OMB minimum categories) was implemented/achieved at each study site, when that occurred at each site, and how that affects the data quality?*

**We appreciate the reviewer pointing out the complexities and limitations of ethnicity classification, both intrinsically and in the context of US health operations. Differences across study sites may reflect variations in regional populations, in how staff communicate with patients to collect ethnicity data and how often it is refreshed, or in data entry, but are less likely to reflect different coding systems. We have expanded on our original sentence to clarify our point that one of the ways DQA is valuable is early identification of issues that will affect study analyses, even if they are not due to technical problems in construction of the analytic dataset.**

*5. Aspects of the discussion really should appear much earlier in the manuscript for readers to understand what is being done and why (e.g., the text at lines 291-296).*

**Thank you for this comment. We have made the following changes to address reviewer concerns:**

- **We have moved the content from lines 291-296 to the Methods. We believed we were summarizing the content of the paper but agree that it would help if instead they were presented earlier.**
- **We moved the Methods section earlier in the paper.**
- **All of the results that are currently in the Discussion section are presented first in the results, whether in a table, figure, or in text.**

*6. There is inadequate information about how data quality issues identified were corrected/resolved by the various institutions. Who assessed whether and how were changes determined to be sufficient to make the data appropriate for subsequent analysis?*

**Thank you for pointing this out. We have addressed this comment by making the following changes:**

- **We have included more information about the purpose of prioritization and how these results were presented to institutions (Methods Section, Subheading: “SSDQA Evaluation”).**
- **In the Results section, we describe more clearly the process of remediation with the sites (Subheading: “Remediation and Improvement”).**
- **We added a new section to the Methods that describe the rounds and purpose of data quality submissions, as well as the process for reviewing comparatively anomalous site values, and documentation of measurable improvement ( “SSDQA Check Sequencing”).**

*7. Fig 8 is not mentioned anywhere in the main text. Did you intend for the reference to “Figure 1” on line 383 to direct readers to Fig 8 instead?*

**We have corrected this error.**

*There are an assortment of writing issues that are confusing and/or distracting and should be addressed with any revisions. These include the following:*

*8. Line 87, p6 refers only to use of data collected for “operational” purposes, but EHR data actually also include data collected for “treatment” and “payment” purposes (see HIPAA 164.502(a)(1)(ii) “for treatment, payment, and healthcare operations”). I would suggest you rephrase to be more precise and accurate.*

**Thank you for noting the potential ambiguity here. We have rephrased to clarify that we intended to include the range of business processes involved in health care delivery.**

*9. Line 99, p6 requires a period after “et al” so it accurately communicates the abbreviation of the latin phrase meaning “and others.” This problem is repeated throughout the manuscript (e.g., Line* *129, p8).*

**We have regularized our use of this abbreviation to include a trailing period.**

*10.  It’s unclear why the words “completeness,” “plausibility,” or “conformance” are italicized in Lines 99-100 p6 and seems unnecessary. Same with “fit-for-use” at Line 101. The italics font does not appear to provide any extra clarity. Same is true elsewhere in the manuscript (e.g., words on lines 130-133, p8). The only place where italicized font seems to provide clarity is when you use it for mentions of “Round 1 DQ” (such as on line 140, p8) and “Round 2 DQ” (such as on line 149, p9). However, you do not use this style consistently (e.g., “Round 2” is not italicized on line 156, p9; inside Table 1; or at lines 272-273, p21). This manuscript overutilizes italics, which is likely to create confusion for readers. For example, why is serum cystatin italicized in some places but not italicized in Fig. 1?*

**We appreciate the feedback about where italics were adding to or detracting from clarity. We have reduced the use of italics throughout, limiting it to introduction of labels.**

*11. For lines 141-145, p8, it is unclear what the numbers in the parentheses of the 10 listed domains mean. Are those references to citations or perhaps some sort of count that relates to the “79 data quality checks” noted in line 141? If citations, there are missing references from the list at the end of the manuscript (which only goes to Ref #24). It appears that they might be counts that sum to 79; however, if that’s the case, the sentence structure is highly problematic/confusing when it continues to include “four check categories” that also have parenthetical numbers listed. Even though the next sentence begins with “as the counts indicate,” the phrasing of the preceding sentence is clear as mud. The same criticism applies to how you’ve structured lines 150-156 on p9.*

**Thank you for pointing out the potential for confusion with references. We have rephrased these sentences to remove the ambiguity, and to separate check data domain and target category more clearly.**

*12. USRDS is never spelled out as the United States Renal Data System. That should be done in the first appearance of the acronym, which seems to be Line 168, p9.*

**We have corrected this.**

*13. “ETL” (e.g., line 281, p22) is a term of art that should be explained for readers.*

**We have expanded the term at its first use, supplementing the more detailed description in Methods.**

*14. The 73-word sentence at lines 190-195 is meandering and confusing. Consider breaking it up for readability.*

**We have corrected this by splitting into 3 sentences, lines 356-362.**

*15. Sometimes you call the first round of data quality assessment “Round 1 DQ” and later “aggregate level round (DQ1)”. Is there a rationale for when you use which descriptor? A similar question applies to reference of “Round 2 DQ”; “row-level phase (Round 2 DQ)” at line 234, p18, and later “record-level round (DQ2)” at line 313, p23.*

**Thank you for pointing this out. We have standardized on DQ1 and DQ2 throughout the manuscript.**

*16. What is the purpose for use of the opaque term “stakeholders” at line 350? Is there a more appropriate and less value-laden term available? For examples, see alternative terms discussed by the CDC at* [*https://www.cdc.gov/healthcommunication/Preferred_Terms.html*](https://www.cdc.gov/healthcommunication/Preferred_Terms.html)

**We had originally used the term preferred by PCORI, this study’s sponsor, to describe the range of interested persons. We agree that usage is evolving, and have shifted to more descriptive language.**

*Other issues that copyeditors should help correct include these:*

*17. It is unclear why the beginning of each paragraph is not indented properly.*

**We have indented the first line of each paragraph by a consistent amount.**

*18. Extraneous space following the hyphen in “healthcare-specific” in the abstract at line 46, p3*

*19. Unnecessary commas following “governance” in line 85, p6; following “overall” in line 110, p7; following “elements” in line 185, p13; following “cohort” in line 189, p14*

**We have amended the text at these locations.**

*Reviewer #2: Your article excellently demonstrates the significance of robust data quality testing, especially in studies making secondary use of clinical data. The systematic approach you implemented for data quality assessment, integrating widely adopted concepts with healthcare-specific evaluation methods, was particularly noteworthy. The two rounds of evaluation you conducted, focusing on both high-level aggregate evaluation and extended row-level data analysis, provided a comprehensive understanding of the data quality issues encountered.*

*The identification of over 100 data quality issues in each round, encompassing various dimensions such as completeness, data model conformance, cross-variable concordance, consistency, and plausibility, is a substantial contribution. Your emphasis on cataloging and prioritizing issues for remediation, leading to the successful resolution of numerous data gaps and extraction errors, is commendable. Moreover, your insights into complexities related to measures of kidney function and their implications for outcome definitions provide valuable insights for future research in this domain.*

*Your study rigorously evaluated the fitness of data for its intended use, and the framework you have developed is both reusable and theoretically robust. The findings from your work not only contribute significantly to addressing data quality issues in healthcare research but also underscore the imperative need for multidisciplinary teams when handling real-world data.*

**Thank you for your investment of time in reviewing the manuscript. We are grateful that you found both our evaluation results and our placing of this work in the wider context of data-intensive clinical research of significant value.**

*Reviewer #3: Summary of the Research and Overall Impression:*

*Razzaghi et al.'s paper, "Systematic Data Quality Assessment of Electronic Health Record Data to Evaluate Study-Specific Fitness: Report from the PRESERVE Research Study," provides a comprehensive exploration of a study-specific data quality framework in the context of a multicenter retrospective study.*

*This work builds upon the author's previously cited manuscript, "Developing a Systematic Approach to Assessing Data Quality in Secondary Use of Clinical Data Based on Intended Use."*

*The paper effectively aligns its abstract and tables with the primary study objective.*

*Strengths:*

*This manuscript offers significant strengths by highlighting the critical role of Data Quality Assessment (DQA) in real-world clinical research, particularly within Electronic Health Record (EHR)-based studies. It vividly illustrates the substantial heterogeneity among sites and inconsistencies across data variables, underscoring the importance of DQA. The incorporation of multiple figures (1-7) and table 2 serves as a compelling showcase of the framework's real-world applicability.*

*Overarching Comment:*

*While the paper successfully applies the framework, there is an opportunity to transition it from being primarily an application piece to a demonstration of the framework's potential generalization across diverse research contexts.*

**We appreciate the thoughtful evaluation driving the comments. Our goals in this study were threefold: 1) to extend the theoretical framework published previously with the practical aspects of implementation, 2) to assess the impact of systematic DQA on conduct of a large clinical study, and 3) to identify lessons learned in the process of application. We agree with the comment that demonstrating reusable aspects of application is valuable to the reader, and have worked to incorporate that through detailed descriptions of check and issue cataloging, reporting guidelines, and approach to remediation, that can be used as a template by other researchers applying similar checks to their studies. We have made revisions throughout to highlight these guides. There are other parts of the reported work that we believe served as a test for the framework’s underlying principles, and are critical to report as part of open science and transparency. While they are built on specific problems encountered, the refinements we identified in this application provide opportunity for us to better move forward with broader generalizability and automation. We hope that the revision balances these goals, to help readers understand not only what to generalize but also what pitfalls to avoid.**

*Minor Weaknesses:*

*- The paper effectively details the application and operationalization of the framework in the methodology section. However, it could benefit from clearer guidance on how readers can replicate the results, particularly given the room for interpretation in check development.*

**Thank you for this comment. We have addressed this concern in several ways:**

- **We have modified the main text of the manuscript to provide more details about application of the checks, and how others may replicate across diverse contexts.**
- **We provide a more detailed example of how an investigator may modify checks based on the framework principles to align with their specific use case.**
- **We have extended Table 2 with more examples of the specifications, showing how the principles of the framework lead to a specification for a check.**
- **We have reformatted Table 1 to be more intuitive and aligned with the framework.**
- **Included in this manuscript are our check catalogs, which show how general properties of checks, including *check type, check category, data quality probe,* and *clinical goal*, find concrete application in a particular study. To illustrate this, we also list the variables that they were applied to, and the column *summary* provides the summary of the applied check. We believe that the level of detail provided in these catalogs facilitate reproducibility across contexts, and we refer to it more explicitly in the main text of the manuscript.**
- **We have placed our check code on GitHub, so that other researchers can review how the principles were applied.**

*- In the authors' original article (citation 23) detailing the framework, they mention “*The framework is open-ended in that it does not fully prescribe a checklist of concrete DQ tests to apply, as achieving this degree of specificity for all cases would result in a highly complex process. Nor, as a conceptual framework, does it create the executable code to evaluate the checks. Rather, the purpose of this framework is to systematically approach a known problem that is often addressed on an ad-hoc basis or as a partial preprocessing step for analyses.”* Although I understand this principle, I would have appreciated it for the authors to delve deeper into the process of check creation and illustrate it more explicitly.*

**We agree with the reviewer and have addressed this with additional detailed text in the Methods section that both describe how checks were developed, as well as examples of how the checks can be extended. We also expanded Table 2 to provide more examples of check creation. We recognize that in the application of our framework, check development using the principles detailed in the paper is not deterministic, but is better described as applying best practices, and describe these more completely. Similarly, we believe the diversity of possible checks makes it infeasible to enumerate all SSDQA applications across diverse contexts. This critical step of evaluating our approach in a particular context helps to identify areas of deep coverage and gaps in our development process. We demonstrate in the paper how one could use this approach and tailor it across study contexts.**

*- Figures 8 and Table 3 require improvement to enhance reader comprehension of such. Figure 8 should more explicitly convey that variable selection guides check categories, types, clinical goals, and data quality probes. In Table 3, grouping Check Type/DQP/DQI per check category would provide better clarity (I understand that clinical goals cannot be categorized in the same way).*

**We have updated Figure 8 (now Figure 1) to emphasize the way in which study variables drive DQ check specification. We have also restructured Table 3 (now Table 1) to avoid giving the misimpression of a hierarchical organization, since these characteristics for a highly connected network**

Stylistic Details:

*Consider relocating the methods section from the bottom to after the introduction to enhance the paper's organization and flow.*

**We had originally placed the Method at the end following the journal’s author guidelines. On reviewing several recently published papers, however, we found that the journal currently publishes manuscripts with the Methods section prior to Results, and agree that this flows better in our manuscript. We have made this change.**

*Other Notes:*

*Questions for the Authors*

*i. The distinction between DQA rounds 1 and 2 not being inherent to the framework and being specific to this study raises questions. Could the authors elaborate on why this was not included in the original framework paper? Additionally, is there a way to systematize the task division for data quality assessment, considering that some studies may require multiple rounds? Providing guidance on how researchers can navigate such implementations would be valuable.*

**Thank you for highlighting this important consideration. Since the initial paper focused on defining the content of tests, it gave less space to factors primarily affecting execution. We have expanded the text on check sequencing in Methods to better articulate pragmatic reasons for use of separate rounds in PRESERVE.**

*ii. The most significant impediment to the generalization of this framework appears to be the development of DQ checks. Could the authors elaborate on potential solutions to automate the development of DQ checks, thus streamlining the framework's application?*

**This is an area of active research, including our own, on the balance between reusable or automatable logic and the need to address specifics of the study. For the current manuscript, we have identified the needs and limitations in greater detail in the Discussion.**

*iii. While the primary objective of this study was not the correction or assessment of DQ checks, it would be beneficial to see more explanation regarding the actions that can be taken when encountering a failed DQ check. Perhaps the authors could consider including a table showing potential actions categorized by different Check types or categories.*

**While it is difficult to lay out a comprehensive set of possible causes for broad classes of DQ issue, we agree that guidance is valuable to make remediation more effective. Following this suggestion, we have added in Supplemental Table 3 a list of common considerations that may be helpful to guide investigation of different issue types. Further, Figure 6 maps data quality outcomes to priority level.**

*iv.  Is it possible to add the number of observations per site. Can the authors also comment on external factors affecting DQA. (i.e. academic vs non-academic centers; presence of informatics team, etc.)?*

**We have added descriptive data about participating institutions to the Results section.**

Recommended Course of Action (require manuscript modification):

*1. Enhance the paper's emphasis on the broader utility of the framework, shifting from primarily an application piece to a demonstration of its generalization potential.*

**We have made a number of modifications throughout to increase discussion of repeatable principles and guidance for SSDQA in other studies (please see above).**

*2. Provide clearer guidance on how readers can replicate the results, especially in the context of check development.*

**Please see above as well.**

*3. Improve Figures 8 and Table 3 to enhance reader comprehension of the check creation process.*

**We have added further explanation to the text and exhibits describing check development (detailed above) in ways that we hope will more fully assist the reader in generalizing to other studies.**

*4. Consider relocating the methods section to after the introduction for improved organization.*

**We have made this change.**

*5. Explore potential solutions for automating DQ check development to facilitate the framework's broader adoption.*

**We have expanded our discussion of reuse in the Methods, and have added text to the Discussion describing that automating check generation is an area of active research.**

*6. Offer more explanations regarding actions to be taken when encountering failed DQ checks, potentially through the inclusion of a table categorizing potential actions by Check types or categories.*

**We have added a Supplemental Table enumerating common considerations when investigating DQ issues.**

*Reviewer #4: Thank you for the opportunity to review the manuscript by Razzaghi et al. The manuscript describes the procedure used to systematically assess the quality of electronic health record data to evaluate study-specific fitness in the PRESERVE study. The aim of the study is to describe this process such that it can be replicated in other clinical studies. The manuscript is clearly written and addresses an important and relevant topic that is applicable to many studies. However, the structure of the manuscript is rather confusing, which makes it difficult to grasp the essence of the study and several points require clarification to make the manuscript more insightful and thereby truly reusable by other studies (one of the main goals).*

*General concerns:*

*1. In general, more concrete explanations and examples are required to understand how the data quality checks were performed. For example, in the Methods (Line 400-401) the authors state that they checked “presence of sufficient clinical information” and “whether clinical information follows expected patterns”. How is ‘sufficient’ defined? What is ‘expected’? It would be helpful if the authors provide several specific examples in the manuscript (e.g. by expanding Table 4, specifying when eGFR would be flagged as ‘anomalous event sequencing’) and provide the entire list of specific checks as a supplement (e.g., additional column in Supplemental Table 2). As an example of what I mean, see the examples provided in Table 1 in the publication by Kahn et al. (10.13063/2327-9214.1244, ref 14 cited by the authors).*

**We appreciate the reviewer’s suggestion that more detailed examples would help the reader, as we include both generalizable principles and specific applications in the paper. We have updated the Methods text to more fully specify how categories such as completeness and plausibility are applied. In addition, we have expanded the former Table 4 (now Table 2) with additional examples and added specific language for each check. Finally, we have added a pointer to the code implementing DQ checks referenced in the catalog in Supplemental Table 2, so that the reader can review the logic in detail. The “summary” column in the catalogs (Supplemental Tables 1 and 2) reflects the descriptions we used to drive development of check code. Table 2 in the paper is expanded to provide more context to a wider audience.**

*2. In addition to the codesets on Github, can the authors share the code used to generate the reports? I couldn’t find this.*

**We have placed the code that implements DQ checks on GitHub for reference as well as the codesets.**

3. I am quite concerned by the remark that many issues could not be resolved because of the absence of a multidisciplinary team (Line 273-276). I don’t see how the quality of clinical data can be assessed without input from clinicians or a broader multidisciplinary team. How does this affect the validity of the approach? Please discuss this limitation in more detail in the Discussion.

**We agree strongly that both informatics and subject matter expertise are essential to understanding data quality. Our intent was to highlight that limitations in study budgets and timelines often constrain the resolution of DQ issues; sites did in fact employ a multidisciplinary approach, but we acknowledge that often times the scope exceeds available capacity. Prioritization based on impact to study analyses is one way to address this gap, but we argue as well for greater attention in study design. We have updated text in Methods and Discussion to frame this more clearly.**

4. The manuscript would benefit from a more strict separation between Results and Discussion. It seems that some results appear for the first time in the discussion (e.g. line 305-306 and line 309, line 314-318, line 323-324, line 326) and that some points are discussed in the results that would be better placed in the discussion (e.g. see comment 3 above).

**Thank you for pointing this out. We have addressed better delineation Results and Discussion sections by reordering sections of text and ensuring we show in the Results anything mentioned in the Discussion. Specifically:**

- **Variation in cohort entry dates (previously lines 305-306) is mentioned in several places throughout the manuscript. Notably, it is mentioned in Table 3 (*variation in time of cohort entry*) and again in Table 4 as an example of an issue for one of the sites.**
- **One site defaulting lab values (previously lines 305-306) is mentioned explicitly in Table 3 (*implausible value detection*).**
- **The impact of clinical coding heterogeneity (previously line 309) is shown in Figure 3.**
- **Checks describing eGFR stability (previously lines 314-318) is described in Table 2 and an example of an anomaly in Table 3 (*event sequencing anomalies*). Importantly, eGFR uniformity of institutions is described in detail on lines 350-362.**
- **Temporal relationships between cohort entry and initiation of antihypertensive medications (previously lines 314-318) is in Figure 9.**
- **Description of clinical correlations, specifically systolic and diastolic blood pressures (previously lines 314-318) is now in Table 2.**
- **Example of comparatively anomalous institutional values mentions variation in specialty utilization (previously lines 314-318) in Table 3 (*variation in clinical utilization*).**
- **The example of one site improving from <5% to 73% of the cohort with a urine protein value (previously like 323-324) has been removed from the Discussion section and moved to Results in lines 408-409.**
- **We show throughout the manuscript examples of anomalies that can be taken into consideration during study analyses, particularly in Table 3.**

*5. Figure 5 and 6 are key figures because they are supposed to show how the DQA process led to higher quality data (one of the main goals of the study). However, both figures are hard to understand and should be improved.*

*a) Figure 5: the goal of this figure is to compare sites before & after remediation; can the authors modify the figure (somehow combine panel a&b) so it’s easier to compare each site before & after. Also, what do these attrition steps on the x-axis represent?*

**We have included descriptions of each attrition step in the legend (now Figure 7) for clarity. We attempted to combine the data into a single graph but this yielded greater complexity of interspersed lines, so we have retained the two-panel approach and aligned the panels in a way that we hope makes it easier to recognize the two sites with critical improvement after DQ testing.**

*b) Figure 6: it is not clear what this figure represents, how does panel B show that data was improved after remediation? Which category is the goal? Why does data disappear from panel A to B, e.g. cbg from site 1 is present in panel A but not in B?*

**We have realigned the figure (now Figure 8) and included additional detail in the legend about the direction of improvement, as well as the retrograde result at one site driven by data governance.**

*Specific concerns, many relating to my general comment of improving the clarity of the manuscript:*

*1. Line 159 + 160: are ‘data quality findings’ and ‘data quality issues’ the same thing?*

**Thank you for pointing this out. Findings are the collective data quality results, and issues refer to specific problems identified.**

*2. Line 162: ‘several institutions did not extract all eligible nephrology specialty visits’: how exactly did this become clear in the report?*

**Thank you for pointing this out Attrition graph updated, and accompanying text.**

*3. Line 163: abbreviation ETL is not defined anywhere*

**We have added this definition.**

4. Line 171: how does Figure 2 ‘highlight the missingness of chronic dialysis code’? This is not clear.

**We have rephrased this to clarify that the focus of what is now Figure 3 is heterogeneity in both code choice and frequency.**

*5. The authors write on several occasions that issues were ‘remediated’ or ‘remediable’ (e.g. line 173, line 213), how can issues be remediated retrospectively in case of missing data? Or was the data available in the hospital systems but not extracted correctly? This should be specified.*

**In this retrospective study, remediation of study data could only occur through correction of ETL errors or discovery of data in source systems; changes in data capture may be possible in a prospective study. We have updated the text to clarify this.**

*6. Figure 2: legend for the color gradient is missing*

**We have added a clearer description of the color scale to the legend for what is now Figure 3.**

*7. Line 191 & Figure 3: ‘only 13% of the potential cohort for one institution had a low eGFR’: the lowest line in Figure 3 at 50% threshold looks higher than 13%.*

**Thank you for pointing out this typographic error. The correct value is 17%. We have also added a horizontal guideline at 25% in what is now Figure 4 to assist the reader.**

*8. Figure 4: please add an informative x-axis label*

**We have corrected this.**

*9. Line 236: why is missingness of variables not critical to key study aims a high priority issue?*

**We assigned the “urgent” priority to missingness in variables that would be needed in most or** **foundational analyses (*e.g.*, eGFR), and “high” to variables that were major components of specific analyses but whose absence would not disrupt other analyses. We have revised the text to clarify this.**

*10. Line 326: ‘We also identified sources of variation that can be accounted for in upcoming main analyses’: what sources of variation?*

**Thank you for raising this. We describe in multiple places where there are sources of variation that may detect an anomaly not in data extraction but instead in other underlying characteristics of the health system or data architecture, such as Examples are listed below:**

- **differences in clinical practices, often reflecting population served, business practices, clinical workflows**
  - **Variation in number of eGFR measurements per person (Table 3)**
  - **Variation in eGFR values (Figure 4)**
  - **Variation in code utilization (Figure 3, Table 3)**
  - **Variation in clinical protocols, where one site may prescribe antihypertensives sooner as a matter of clinical practice (Figure 9)**
- **differences in data architecture**
  - **Visit type classification (Table 3)**
  - **Data availability (Table 3)**
  - **Availability of specialty data (Figure 7)**
  - **Representation of lab metadata/results (*e.g.*, urine proteins) (Figure 5)**

**Because these are not *necessarily* technical problems (though availability of specialty data was able to be resolved), they need to be addressed by appropriately designed and robust analyses. We have attempted to clarify this reasoning in the text.**

*11. Line 421-422: ‘we adopted software development best practices such as …’: this sentence needs a reference*

**We have added references that describe some of the practices used.**

*12. Ref 20 is the same as Ref 9, this should be fixed.*

**Thank you for pointing this out. We have resolved the duplicate entry.**
