# Supplementary material for: Systematic data quality assessment of electronic health record data to evaluate study-specific fitness: Report from the PRESERVE research study
Source: PLOS Digit Health. 2024 Jun 27;3(6):e0000527. doi: 10.1371/journal.pdig.0000527 (PMC11210795; doi:10.1371/journal.pdig.0000527)
Supplement: Attachment — Submitted filename: reviewer_comments.docx [file pdig.0000527.s006.docx]

PDIG-D-23-00422R1

Systematic data quality assessment of electronic health record data to evaluate study-specific fitness: report from the PRESERVE research study

PLOS Digital Health

Reviewer #5: The author amended the manuscript based on comments from the previous reviewers.

There are no comments from my site.

the manuscript is well prepared and provides readers with clinically valuable information.

Thank you for the comment and observation. We have attempted to address and incorporate peer-review comments wherever possible to strengthen the paper.

Reviewer #6: The manuscript effectively demonstrates the application of systematic data quality assessment in a real-world research setting. The thorough evaluation of fitness for study-specific requirements is commendable and highlights the importance of addressing data quality issues in secondary analyses of clinical data.

Thank you for the review and commentary. We address all critiques below.

Besides, the manuscript provides a comprehensive analysis of the application of a study-specific data quality assessment (SSDQA) framework in the context of the PRESERVE study. However, it has several limitations and areas for improvement. These include limited generalizability, resource intensiveness, incomplete evaluation, lack of longitudinal analysis, and absence of comparative analysis.

We agree with the reviewer that there are limitations to this study, which we list in our Discussion section. Widely adopted practices for study-specific data quality assessment have not been established in the realm of multi-institutional EHR research. Most studies test missingness and basic variable plausibility, and all too often detect data quality problems only when analyses are disrupted. We evaluated against this background, and have tried to make this clearer in the manuscript. The research we conducted needed to extend existing frameworks, create reusable processes and tools (recognizing that generalizability of study-specific testing has to be at one level of indirection), evaluate the outcomes, and provide useful content for the PRESERVE study. We also more fully address relationship to other proposed methods for SSDQA, knowing this is an active field of research, if not yet one where practice has been established. We also agree that this complete process was intrinsically resource intensive, though much of that investment was into the study of SSDQA itself and the development of reusable infrastructure. We believe that future applications will be more efficient, benefiting from the time investment of our work.

We have added text to expand on our comparison to existing PCORnet data quality testing and on the relationship of our work to prior proposals for SSDQA design. We have tried to balance in the manuscript the framework’s broad constructs with our specific application in the area of kidney disease. We hope this has yielded a range of information useful to the reader. We believe it would overburden the current study to add an extensive comparison of our method to other approaches proposed as research in themselves. We do agree with the reviewer that an appropriate future step could be to compare across multiple studies our approach to loosely defined *ad-hoc* exploratory analyses, which is the current standard in the field, in order to generate a broader evidence base.

Throughout the manuscript, we have tried to clearly describe how our methods can be generalized to other applications or contexts (particularly the Check Construction, Check Sequencing, and Resolution and Improvement sections of the Methods and from paragraph 3 onward of the Discussion). Further, we share comprehensive check catalogs in our supplemental materials that place our specific checks in a generalizable framework, and include text in the manuscript describing how the PRESERVE data quality checks can be modified for other studies, leveraging our systematization and terminologies (*e.g.* Check Development section in Methods describing how a check can be applied to a Diabetes study, Discussion paragraph 3). While we agree that future work should build on these tools to further increase generalizability, our study is the first to our knowledge to simultaneously apply the principles of an SSDQA framework and provide concrete resources with examples for reusability.

The manuscript lacks a thorough discussion of existing research on SSDQA frameworks in the context of observational studies using electronic health record (EHR) data. A more comprehensive review of the literature could help contextualize the study's contributions and identify gaps in current knowledge or methodologies. Comparing the SSDQA framework with existing approaches in the literature would help evaluate its novelty and effectiveness.

Validation and benchmarking of the framework against established standards or best practices in data quality assessment would enhance the credibility and reliability of the findings. Future directions for the SSDQA framework could be discussed, including potential extensions or refinements, opportunities for further empirical testing, and cross-disciplinary insights from related disciplines such as epidemiology, biostatistics, and health services research.

Thank you for this suggestion. While it was not our goal to provide a systematic literature review of existing data quality frameworks, we have included in the Introduction a summary of the limitations of Kahn’s widely adopted harmonized data quality terminologies, existing network data quality programs, and previously published SSDQA frameworks (*cf.* lines 100-142 of tracked version). Similarly, we include limitations of data quality assessment in the statistical literature in the same section (*cf.* lines 122-128 of tracked version). In a later section, we describe how our work complements current practice in statistics and health services research (*cf.* lines 580-592 of tracked version). Finally, we included in the discussion of the paper additional information on next steps, focusing on automation and accessibility (*cf.* lines 595-600 of the tracked version).

Incorporating insights from related disciplines like epidemiology, biostatistics, and health services research could enrich the discussion and broaden the study's relevance to a wider audience. Addressing these limitations could enhance the manuscript's contribution to the field by providing a more nuanced understanding of the SSDQA framework's effectiveness and applicability in observational health data analysis.

Thank you for this suggestion as well. Throughout the paper, we have discussed the utility of the framework in informing study decisions, particularly for disciplines like epidemiology, biostatistics, and health services research. For example, Table 3 demonstrates how specific data quality issues detected in our work have analytic implications. While we focus on the PRESERVE use-case to provide concrete instances, we synthesize the information to highlight its generalizability to other studies. We have also added text that describes how this framework is designed to complement current advances in biostatistics and epidemiology in accounting for data quality limitations in EHR data (*cf.* lines 580-592 of the tracked version).

Reviewer #7: The manuscript is of a outstanding importance for the field and it highliths the need for human activity in data quality assessment rather then only relying on the so deified anrtificial intelligence and machine learning. The authors have made a well panned and rigourous work which embasis other researcher to follow their paht and previous efforts to improve data quality assessement for the secondary analysis (and primary, in some cases too) studies that are so necessary and important both beacuse the data are available and low cost and because provides rech information and allows great researchs to be performed with.

Nevertheless, there are some minor issues for the authors to address, presented by section and lines:

Abstract:

Lines 52 and 57: The term “remediation” induces the readers to infer that the procedure to improve data quality does not correct the issues, but provides some kind of “non-conventional strategy” to make data usable. Consider using “resolution” or another synonym.

We have replaced “remediation” with “resolution” or similar language.

Line 64: Consider exchanging “backed by” for relies on/ conducted following.

We have made this change.

Line 64: The sentence starting with “Major data” is confusing. Are the authors trying to say that “major data quality issues addressed in the conduction of the process would have otherwise…”?

Thank you for pointing out this ambiguity. We have corrected the sentence.

Introduction:

Line 114: The term “programs de novo” needs to be explained once it is not usual or commonly used for this purpose, thus possibly misleading the interpretation.

We have rephrased this text to be clearer.

Line 115: A brief explanation on what Study-specific Data Quality Assessment is crucial for the manuscript comprehension.

We have expanded this sentence in a way we hope clarified the connection to other text around it (*cf.* lines 113-114 in tracked version).

Line 116: This paragraph states the aims and part of the methods. Consider presenting only the background and the directions for the study background construction.

We appreciate the overlap noted by this comment, and have moved and rephrased text to more cleanly place in the introduction aspects of the PRESERVE study that motivated our work, and in the methods description of the study infrastructure.

Line 130: This paragraph would be better placed previously to the one above, which might be excluded or rewritten in a less detailed form since it presents information that will be again presented by the authors in the following section.

We hope that the reframing of the prior paragraph makes the flow of this section smoother.

The authors do not present and overview of what PCROnet is, so the comprehension of the methodology and study conduction get hard for the readers that are not familiarized to it.

We have added a brief description of PCORnet at this point, and further detail on relevant aspects of PCORnet later in the text.

Material and Methods:

This section lacks an introduction, and this issue could be addressed by replacing the paragraph starting in line 116 to this section.

Please see above. We have moved content from the former line 116 to the Methods as introductory text, under the subheading, *Study Setting*.

Line 157 and 158: There is no Figure 1 in the manuscript.

Figure 1 as submitted is a block diagram of the data quality development process.

Line 215: The sentence starting with “to address” if confusing. Did the authors develop a software? Did they followed a software development guideline/procedure to perform the study?

We have rephrased this sentence to indicate the effort invested to produce more reusable checks, compared to the one-off coding often done as part of DQA.

Line 238: What is PRESERVE CC? It is only presented in the next paragraph, so this explanation needs to be moved to this sentence.

We have moved the definition to the first use in this sentence (*cf.* line 293 of tracked version).

Line 243: What is a SAS query? Is it related to SAS software?

We have expanded the text here to clarify that SAS is the software that is the current PCORnet^®^ standard, and was used when interacting with PCORnet^®^ sites (*cf.* lines 298-306 of tracked version).

Results:

Lines 313 to 315: the sum of the percentages results in 101%.

This results from rounding of fractional percentages. In order to rectify the total, we have decreased the value least affected by rounding.

Line 413: What is Area Deprivation Index? Although the authors mention the reference for it, a brief explanation/presentation is required.

We have added a brief description of the ADI to the sentence.

Reviewer #8: The manuscript presents valuable insights into systematic data quality assessment in the context of electronic health record data analysis. However, several key areas require clarification and elaboration to enhance the reproducibility and credibility of your findings. Specifically,

Abstract:

1-      The abstract mentioned on line 53 “over 100 quality issues”, this statement lacks specificity and the method used by the authors is not specifically mentioned.

We have rephrased this sentence to add precise counts and bring forward the descriptions of check categories.

2-      It was suggested on line 65 the need for muti-disciplinary teams to address data quality issue, how? Why? This statement appears a bit general and could be to some extent be ambiguous.

We have rephrased in a way we hope adds greater specificity.

Introduction

1.      Line 84, the citation needs to precede the end of the statement.

We have corrected the error in punctuation.

2.      Line 115, define the acronym DQA.

In response to other reviewer comments, we have rephrased this sentence so that the abbreviation is resolved.

Results

1.      Line 140, define DQ.

We have compared the line numbers cited by reviewer #8 to prior versions of the manuscript, and it appears that the reviewer had the original version of the manuscript, not the most recent revision. We have located a first use of “DQ” at line 245 of the last revision (now line 302 in the tracked version), and glossed it for consistency.

2.      Section starting from Line 207: While the text discusses data quality remediation efforts, it provides limited insight into the specific actions taken to address identified issues. It mentions that some issues were remediated by affected institutions, but it does not elaborate on the nature of these remediation efforts or their effectiveness.

We hope that the additional information and reordering of sections in the prior revision substantially address the reviewer’s comment. Nonetheless, we have also added more specific language to the resolution section of the manuscript. The revisions included references to figures demonstrating the improvements.

Discussion

1.      How is the present SSDQA framework validated and applicable to any other research study? (What is the empirical evidence provided by the authors)

This is a central goal of our manuscript; we are grateful to the reviewer for placing focus on it. We have tried to be more explicit in several areas. We added detail on how this type of DQA relates to current standard practice, including PCORnet Data Curation, and how that applies across studies (*cf.* lines 100-162 of tracked version). In addition, we highlight how the specific checks here apply methods that are applicable across studies: while PRESERVE-specific testing may focus on kidney-related variables (and directly generalize to any kidney-related study), it also provides structure for assessing laboratory testing, medication usage, time-dependent changes, etc., and therefore generalizes to many study types with appropriate substitution of variables. Further, we share comprehensive check catalogs in our supplemental materials that place our specific checks in a generalizable framework, and include text in the manuscript describing how the PRESERVE data quality checks can be tailored for other studies, leveraging our systematization and terminologies (*e.g.* Check Development section in Methods describing how a check can be applied to a Diabetes study, Discussion paragraph 3). We have also highlighted that check results were validated by Coordinating Center review, study team consultation, and joint review with teams at the sites that generated data, in order to provide additional empirical support beyond the data presented directly in the manuscript.

2.      What are the costs and benefits associated with the implementation of this framework which happens to be resource-intensive?

We have tried to highlight through the manuscript the different balances associated with *studying the framework itself*, which we present here and involved additional work on refinement and evaluation, and *using the framework*, which can focus more on applying reusable resources and integrating into study operations. We have added detail on this topic to the latter part of the Discussion. We have also noted that the cost/benefit balance is not limited to the SSDQA “phase” of a study. First, many of the issues detected by increased attention to SSDQA would have become apparent later in the study in much more disruptive ways (*e.g.* requiring regeneration of data late in the study). Second, effort invested to resolve problems detected through this investment, if applied via research networks or data warehouses, will directly benefit other studies using the same data elements. Third, much of SSDQA is learning about the data to make better informed decisions about analyses (*e.g.,* defining variables critical for the study).

Methods

1.      How many institutions participate to PRESERVE study? (Lines 367-431)

This information was added as part of the prior revision and is present in the Methods.

2.      What are the specific criteria used to assess data quality? What are the thresholds for determining good-quality data? What is the prioritization process (Lines 432-69)

The revision added detail in this section (SSDQA Execution) and the next (SSDQA Prioritization) on the identification and prioritization process, and we have added further explanation about criteria for issue identification.

3.      How is the privacy of patient information protected?

We have added a description of data security practices to the Methods section.

4.      The method does not mention the validation procedure to assess the accuracy and reliability of the SSDQA. We suggest the author integrate this section into their manuscript.

We have updated text describing process for review both within the Coordinating Center (when data were available there) and with teams at the originating institutions to assess the accuracy of the results. In the Resolution and Improvement section of the Results, we also note which issues were valid and improved, which were valid and intrinsic to the source data (which we considered valid because even if not repairable they may affect study analysis planning), and which could not be validated.
